# Use of Food Practices by Childcare Staff and the Association with Dietary Intake of Children at Childcare

**DOI:** 10.3390/nu7042161

**Published:** 2015-03-27

**Authors:** Jessica S. Gubbels, Sanne M.P.L. Gerards, Stef P.J. Kremers

**Affiliations:** Department of Health Promotion, NUTRIM School for Nutrition and Translational Research in Metabolism, Maastricht University, PO Box 616, 6200 MD Maastricht, the Netherlands; E-Mails: sanne.gerards@maastrichtuniversity.nl (S.M.P.L.G.); s.kremers@maastrichtuniversity.nl (S.P.J.K.)

**Keywords:** childcare, daycare, dietary intake, feeding style, nutrition, pre-school child

## Abstract

The study explored the associations between various childcare staff food practices and children’s dietary intake at childcare. A total of 398 one- to four-year-old children and 24 childcare staff members from 24 Dutch childcare centers participated in the study. Children’s dietary intake (fruit, vegetable, sweet snack, savory snack, water, and sweet drink intake) at childcare was registered on two weekdays, using observations by dieticians and childcare staff. Thirteen childcare staff practices were assessed using questionnaires administered by dieticians. Data were analyzed using multilevel regression analyses. Children consumed relatively much fruit and many sweet snacks at childcare, and they mainly drank sweet drinks. Various staff practices were associated with children’s dietary intake. When staff explained what they were doing to the children during food preparation, children ate significantly more fruit. Children ate less sweet snacks when they were allowed to help prepare the meals. When staff encouraged children to continue eating, they ate more vegetables. In conclusion, the study showed the importance of childcare staff food practices for children’s food intake at childcare. More research is needed to examine the specific conditions under which food practices can have a positive impact on children’s dietary intake.

## 1. Introduction

Although the prevalence of childhood obesity appears to be stabilizing in various countries, it remains high and an important public health issue [1]. In 2010, at least 42 million children under the age of five were overweight [2]. Childhood overweight poses children at risk for several severe chronic conditions such as type 2 diabetes mellitus and cardiovascular diseases [3], as well as for overweight or obesity during later life [4].

Over half of European toddlers attend childcare or pre-school education facilities [5]. In the Netherlands, 46% of the children below 3 years, and 86% of the children between 3 years and mandatory school age (five years) made use of formal childcare in 2013 [6]. Childcare use has been found to be associated with an increased overweight risk throughout childhood (e.g., [7,8,9]). In addition, children attending childcare often do not meet dietary intake recommendations, consuming excessive amounts of energy [10,11], fat [11,12], saturated fat [12,13] and sweets [11], and insufficient amounts of fruit [10,13], vegetables [10,11,13,14], and dietary fiber [10,15].

Differences between children’s dietary intake can be partially attributed to the childcare center the child is attending [16]. In view of the increasing use of childcare, various authors have called for increased attention for the influence of childcare practices on children’s dietary intake (e.g., [10,17,18]). An important social environmental influence on children’s dietary intake at childcare is staff behavior. As regards the *use* of food practices by childcare staff, previous research has shown that staff uses various favorable practices: they often eat or sit together with the children at the table [19,20,21], and regularly talk with the children about foods and (healthy) eating [19,21,22]. Less favorable practices reported to be used by childcare staff include that few references to internal hunger or satiation cues are made [19,23]. However, overall, there are large differences between centers with regard to the use of most food practices [19,20,21,22,23,24,25,26].

Few studies have examined the *association* between childcare staff food practices and children’s dietary intake [27,28,29,30]. This is a striking contrast with the massive number of studies that have looked into the effects of parenting practices (e.g., [31,32,33,34]). Hughes and colleagues [27] examined the influence of staff feeding style (indicating the general pattern of food practices used by childcare staff) on the dietary intake of three- to five-year-old low-income children. They found that an indulgent feeding style, in which children were for instance frequently offered multiple servings, was positively associated with vegetable consumption. Childcare staff eating together with the children and staff eating unhealthy food in front of the children have been found to be associated with increased intake of the food served [28]. This could be either positive in the case of fruit and vegetables, or negative in the case of unhealthy foods such as biscuits or sweets. When trying to get a pre-schooler to taste a new fruit or vegetable, offering a reward, insisting that the child would try a bit and offering the child a choice between several new foods, have been found to be effective practices [30]. In contrast, staff modeling by eating the new food in front of the child and mere exposure to new foods, were less effective [30]. Enthusiastic modeling by staff (verbally confirming that the new food they were eating tasted good) has been found to increase food acceptance among pre-schoolers [29]. In line with this, staff talking about healthy foods with the children has been found to be associated with increased dietary fiber intake, while using food to control behavior has been found to be associated with decreased fiber intake [28]. However, these previous studies have mostly been conducted in small samples [28,29,30], while examining a limited number of staff practices (e.g., only modeling [29]). In addition, based on the very limited number of previous studies, firm conclusions regarding the effects of staff’s practices cannot be drawn yet.

In order to increase insight in childcare staff’s role in young children’s dietary intake, the aim of the current study is to explore the use of various childcare staff food practices and children’s dietary intake (fruit, vegetables, sweet snacks, savory snacks, water and sweet beverage intake) at Dutch childcare centers. In addition, the associations between staff food practices and children’s dietary intake are examined.

## 2. Materials and Methods

### 2.1. Childcare System in the Netherlands

Dutch children normally start school at age 4, and from age 5 on they are obligated to start school. Children can attend center-based pre-school childcare from 0 to 4 years in the Netherlands. Childcare cenre sizes vary greatly, but very small centers with only one or a few groups of children are also common. Some, but not all, childcare centers are affiliated with larger regional of national childcare organizations. Childcare costs are paid by parents, but they can apply for funding to the Dutch government. How much funding parents receive is dependent on factors, such as their income, the number of hours they work, and how many children they have in childcare.

In the Netherlands, children attending childcare generally have three eating opportunities at childcare: a morning snack, a lunch (often consisting of sandwiches), and an afternoon snack. These foods are usually provided by the childcare centers. Most Dutch childcare centers do not provide a warm meal, and in most cases the meals are prepared by the childcare staff themselves, not by external caterers.

### 2.2. Respondents and Procedure

Childcare centers distributed throughout the Netherlands were approached to participate in the current study. The first 25 childcare centers that agreed to participate were included in the study. All parents of the children aged 1 to 4 years old from these childcare centers were invited to participate. Infants were not included in the study, because their dietary pattern is distinctly different from the dietary pattern of toddlers, as they mainly consume milk feedings. In total, 556 children participated. The number of participating children per center ranged from 2 to 45. All parents of participating children provided written informed consent, and reported children’s age (rounded off to whole months) and gender. The data collection period ranged from August to November 2013. Children’s dietary intake at childcare and the childcare practices were registered on two weekdays within one week, using observations and questionnaires administered by two dieticians. As the current research was observational and measurements were non-invasive, it complied with the Dutch ‘Medical Research Involving Humans Act’ (in Dutch: ‘Wet Medisch-Wetenschappelijk Onderzoek met Mensen’), and, thus, did not need explicit approval by an Ethics committee.

### 2.3. Dietary Intake

Childcare staff was instructed by one of two trained dieticians to register the all dietary intake at childcare, of each of the participating children separately on a poster. On the poster, a partially pre-coded dietary record was printed, providing a list of the most common products consumed at each eating opportunity. For instance, for the morning and afternoon snack, a list of sweet snacks (e.g., biscuits), beverages (e.g., water, juice) and fruits (e.g., apple, pear) commonly consumed at snack times in the Netherlands was provided. In addition, for each eating opportunity there was space to register any other consumed products which were not on the standard list. For each participating child there was a separate column on the poster where the intake could be registered. Childcare staff was asked to specify the *type* of product (e.g., whether the milk product consumed was skimmed milk, semi-skimmed milk, whole milk, chocolate milk, butter milk or yogurt drink), the *unit* (e.g., whether it was a cup or a bottle), and the *amount* (*i.e.*, number of units).

Childcare staff registered the morning snack time of the first observation day together with the dietician. At this point the childcare staff received detailed instructions from the dietician on how to register the dietary intake. In addition, any additional ambiguities from the childcare staff members were explained by the dietician. The rest of the eating opportunities on both observation days (*i.e.*, the morning snack, lunch, and afternoon snack) were registered on the poster by the childcare staff.

An additional questionnaire was filled out by the childcare staff together with the dietician to provide additional information regarding the meals and foods offered at the childcare center, such as the standard portion size used for certain products (how many mL were in the cups used) and the type and brand of certain products (e.g., whether regular or low-fat margarine was used and which brand).

### 2.4. Childcare Staff Food Practices

On the first measurement day, childcare staff filled out a questionnaire regarding the food practices that were used at childcare. This questionnaire was filled out together with the dietician. Previous to filling out the questionnaire, the dietician observed one or more eating opportunities at the center, in order to be able to reflect on these observations when filling out the questionnaire.

Table 1 shows an overview of the concepts assessed in the practices questionnaire. The items of the questionnaire were based on previous studies regarding childcare practices [19,27] and the Environment and Policy Assessment and Observation (EPAO) instrument [35]). The concepts assessed were child involvement in food preparation (2 items), meal time practices (4 items), food rules (2 items), modeling (3 items), instrumental feeding (1 item) and mealtime discussions (1 item; see Table 1).

**Table 1:** Childcare staff food practices assessed in the questionnaire for childcare staff.

Category	Concept	Item	Answering Scale ^a^
Child involvement	Child involvement in food preparation	(Older) children are allowed to help with the preparation/serving/clearing of the food (e.g., setting/clearing the table/preparing a sandwich)	A
	Staff explains food preparation	Staff tells children what they do during the preparation/serving/clearing of the food (e.g., ‘I’m now going to pare the apples’)	A
Meal time practices	Encouragement to eat	Staff encourage children to continue eating (e.g., plate must be cleared, ‘finish your cookie’)	A
	Giving food without asking	Staff provides the children food without asking whether the child wants it	A
	Encouragement new foods	Staff encourages children to try new or less favourite foods	A
	Accepting individual intake differences	Staff accept differences in dietary intake behavior between children (e.g., not rushing a slow eater to eat more quickly)	A
Food rules	Rules about order of eating	Are there any rules about the order in which foods have to be consumed?	B
	Rules about amount of food	Are there any rules regarding the maximum amount a child is allowed to eat or drink? (e.g., a maximum number of slices of bread)	B
Modeling	Staff eat together with children	Staff eats together with the children	A
	Staff eat the same food as the children	Staff eats the same food as the children (e.g., also preparing sandwiches at lunch, also eating fruit as a snack)	A
	Frequency staff consume unhealthy food	Staff eats unhealthy food (e.g., sweets, snacks) in front of the children	A
Instrumental feeding		Staff uses food to control behavior (e.g., (threatening) to take food as punishment, promising/giving snacks for good behavior)	A
Mealtime discussions	Talking about healthy food	Staff talks about healthy foods with the children (e.g., which vegetables they like)	A

^a^ Answering scales: A: 5-point Likert scale ranging from never (1) to always (5); B: No (0) *vs*. Yes (1).

### 2.5. Processing of Dietary Intake Data

Only children for whom complete dietary intake data were available (for both measurement days) were retained in the analyses. Of the 556 children participating in the complete study, 398 (71.6%) provided complete dietary intake data on both measurement days. These children were included in the final analyses. For one childcare center, there were no children with complete dietary intake data on both days, resulting in a final sample of 24 childcare centers. Incomplete dietary intake data were the result of missing observations for one or more eating opportunities, for instance because the child was absent on one of the two measurement days.

The observed dietary intake data of the children were entered by dieticians (different dieticians than the ones instructing the childcare staff during the measurements) in the program FoodFigures (WebArchitecten, 2013) for each eating opportunity separately (morning snack, lunch, afternoon snack). The consumed amounts reported by childcare staff were converted by this program into weight and volume using the procedures on measures and weights of the Dutch nutrient database [36] where necessary (e.g., using a standardized weight for a slice of bread). The average intake per day of the following food groups was calculated: fruit (g), vegetables (g), sweet snacks (g; including sweets (e.g., jelly candy, liquorice, marshmallows), chocolate, cookies (e.g., biscuits, butter cookies) and pastry (e.g., cake, sweet pie)), and savory snacks (g; including salty snacks (e.g., potato chips) and fried snacks (e.g., fried meats)), water (mL) and sweet drinks (mL; including sugar-sweetened beverages and juices).

### 2.6. Data Analyses

All analyses were conducted using SPSS 20.0. *p*-values < 0.05 were considered statistically significant. Descriptive statistics were used to explore children’s gender and age, dietary intake at childcare and childcare food practices (see Table 1). As intake of water and savory snacks was at childcare very low in the current sample (2.0% and 2.2% of the children, respectively), there was insufficient variation of these variables. Water and savory snack intake were therefore not included in any further analyses.

Multiple backward multi-level linear regression analyses were conducted to examine the associations between staff’s practices and children’s dietary intake, corrected for children’s background variables (age and gender), which dietician instructed the childcare worker, and the nesting of children within childcare centers. In these backward multi-level regression analyses, the staff practices were deleted one by one from the analyses in order of their significance, starting with the least significant practices, until only practices for which the *p*-value was <0.10 were left in the model. Children’s age, gender and the dietician were retained in all models.

## 3. Results

A total of 398 children from 24 childcare centers were included in the final sample. There were 179 girls (49.2%) and 185 boys (50.8%), with 34 children for whom the gender was unknown due to missing data. The mean age of the children was two years and three months (standard deviation (SD) = 10 months).

### 3.1. Dietary Intake

Table 2 shows the average dietary intake of the children at the childcare center per day. Children consumed mainly fruit and sweet snacks at childcare, and they mainly drank sweet drinks. Only 2.0% of the children drank water and only 2.2% of the children ate savory snacks at childcare (results not tabulated).

**Table 2:** Average dietary intake by toddlers at childcare.

	Mean Dietary Intake (SD)			
	Total *N = 398*	At Morning Snack *N = 398*	At Lunch *N = 393*	At Afternoon Snack *N = 376*
Fruit (g)	93.4 (46.6)	57.2 (46.5)	3.2 (12.7)	35.0 (45.9)
Vegetables (g)	9.6 (19.5)	0.0 (0.3)	3.9 (10.8)	4.5 (14.4)
Sweet snacks ^a^ (g)	11.2 (11.0)	5.9 (8.3)	0.8 (2.5)	4.7 (5.8)
Sweet drinks (mL) ^c^	265.3 (135.5)	122.7 (71.0)	17.6 (44.7)	133.4 (76.2)

Notes: Water and savory snack intake are not depicted, as they were consumed by only 2.0% and 2.2% of the sample, respectively. g, grams; mL, millilitre; SD, standard deviation. ^a^ Including sweets, chocolate, cookies and pastry; ^b^ Including salty snacks and fried snacks; ^c^ Including soft drinks and juices.

### 3.2. Childcare Staff Food Practices

Table 3 shows the use of food practices by childcare staff. Thirty-three percent of the childcare centers indicated that children were never involved in food preparation, while another 33% indicated that children were always involved. More than half of the staff (54.2%) often or always explained to the children what they were doing when preparing food.

**Table 3:** Reported use of childcare staff food practices (*N* = 24).

Category	Concept	*N* (%) ^a^				
		*Never*	*Seldom*	*Sometimes*	*Often*	*Always*
Child involvement	Child involvement in food preparation	8 (33.3%)	2 (8.3%)	3 (12.5%)	3 (12.5%)	8 (33.3%)
	Staff explains food preparation	3 (13.0%)	1 (4.3%)	6 (26.1%)	7 (30.4%)	6 (26.1%)
Meal time practices staff	Stimulation to eat	1 (4.3%)	1 (4.3%)	6 (26.1%)	12 (52.2%)	3 (13.0%)
	Giving food without asking	11 (47.8%)	7 (30.4%)	3 (13.0%)	2 (8.7%)	0 (0.0%)
	Encouragement new foods	0 (0.0%)	3 (13.6%)	4 (18.2%)	8 (36.4%)	7 (31.8%)
	Accepting individual intake differences	0 (0.0%)	1 (4.5%)	4 (18.2%)	11 (50.0%)	6 (27.3%)
Modeling	Staff eats together with children	2 (8.3%)	1 (4.2%)	2 (8.3%)	5 (20.8%)	14 (58.3%)
	Staff eats the same food as the children	3 (12.5%)	0 (0.0%)	6 (25.0%)	5 (20.8%)	10 (41.7%)
	Frequency staff consumes unhealthy food	17 (73.9%)	4 (17.4%)	1 (4.3%)	1 (4.3%)	0 (0.0%)
Instrumental feeding		15 (65.2%)	5 (21.7%)	3 (13.0%)	0 (0.0%)	0 (0.0%)
Talking about healthy food		3 (13.0%)	1 (4.3%)	11 (47.8%)	8 (34.8%)	0 (0.0%)

^a^ Total N deviates from *N* = 24 due to missing values. Percentages reflect the valid percentages.

During meals, 65.2% of staff indicated that they often or always pressured children to eat, e.g., by pushing children to clean their plate. However, the majority (77.8%) of the childcare staff never or seldom gave children food without asking them whether they wanted it, and most (77.3%) often or always accepted individual differences in intake. Most staff (68.2%) often or always encouraged children to try new foods.

Most staff often or always ate together with the children (79.1%), and ate the same food as the children (62.5%). Most of them never ate unhealthy foods in front of the children (73.9%). The majority (86.9%) never or seldom used instrumental feeding. Talking about healthy food was done sometimes or often by 82.6% of staff.

Most childcare centers had rules about both the order in which foods and drinks had to be consumed (22 childcare centers, 91.7%) as well as the amount of food that the children were allowed to eat (23 childcare centers, 95.8%; results not tabulated). The most common rule regarding the order in which children had to eat was that they first had to eat a sandwich with a savory filling (e.g., cheese, meat products) before they could have a sweet filling (e.g., chocolate spread, jam), which applied by 75.0% of the childcare centers. Other frequently reported rules regarded eating bread crusts first before getting a new sandwich (20.8%), and the order of drinking and eating during lunch (16.7%; e.g., first eating, then drinking; results not tabulated). Rules regarding the amount that children were allowed to consume, most often regarded the maximum number of slices of bread during lunch (ranging from 2 to 4), and the maximum number of sweet beverages (ranging from one a day, to two per eating opportunity *i.e.*, six per day; results not tabulated).

### 3.3. Association between Childcare Food Practices and Children’s Dietary Intake

Table 4 shows that when staff explained to the children what they were doing during food preparation, children ate more fruit (B = 10.16, *p* < 0.01). When staff encouraged children to continue eating, children ate more vegetables (B = 6.11, *p* < 0.01). When children were allowed to help prepare the food (e.g., setting the table; B = −1.85, *p* < 0.05), and when staff gave children food without asking them whether they wanted any (B = −3.86, *p* < 0.05), the children ate less sweet snacks. If staff ate together with the children, children ate more sweet snacks (B = 2.30, *p* < 0.05). There was a borderline significant association between staff eating together with the children, and children drinking less sweet drinks (B = −35.17, *p* < 0.10).

**Table 4:** Association between childcare food practices and children’s dietary intake (*N* = 398).

	Children’s Dietary Intake			
**Childcare food practice**	Fruit (g)	Vegetables (g)	Sweet snacks (g)	Sweet drinks (mL)
	B (*p*)	B (*p*)	B (*p*)	B (*p*)
Child involvement in food preparation	^a^	^a^	−1.85 (0.041)	^a^
Staff explains food preparation	10.16 (0.004)	^a^	^a^	^a^
Stimulation to eat	^a^	6.11 (0.007)	^a^	^a^
Giving food without asking	^a^	^a^	−3.86 (0.037)	^a^
Staff eats together with children	^a^	^a^	2.30 (0.032)	−35.17 (0.088)

Notes: Results of the backward multilevel regression analyses, adjusting for child gender and age, dietician, and the nesting of children within childcare centers. B = Regression coefficient, *p* = *p*-value. The following practices were not significantly associated with any of the dietary intake variables: Encouragement of new foods, Accepting individual intake differences, Staff eats the same food as the children, Frequency staff consumes unhealthy food, Instrumental feeding and Talking about healthy food. ^a^ Variable deleted from final model because the *p*-value was >0.10.

## 4. Discussion

The current paper examined the use of food practices by childcare staff, children’s dietary intake from various food groups at childcare, and the association between both. Children consumed mainly fruit, sweet snacks and sweet drinks at childcare, and little vegetables, savory snacks and water. These results are very similar to the findings of a previous study with regard children’s dietary intake at Dutch childcare centers [10]. Additionally, other studies show relatively high fruit consumption and low vegetable consumption at childcare [14,37].

Interestingly, there were some large differences between the practices with regards to the consistency of their use across childcare centers. For instance, there were large between-center differences when it came to for instance child involvement in food preparation and whether staff explained what they were doing when preparing food. In a substantial number of centers these practices were never or seldom used. On the other side, there also were a substantial number of centers in which these practices were used often or always. The use of other practices was more consistent. Childcare staff consistently reported never or seldom using instrumental feeding (using food for reward or punishment), with only a few (13%) who reported using it sometimes. None of them reported often or always using instrumental feeding. Other food practices that were used quite consistently were the encouragement of trying new foods, accepting individual intake differences and staff eating together with the children. These practices were used often or always in most centers. Giving children food without asking them whether they wanted it and the consumption of unhealthy food by staff in front of the children reportedly happened never or seldom in most centers. In line with the current study, Sisson and colleagues also reported consistently high use of encouragement to try new foods, and low use of instrumental feeding and eating unhealthy food in front of the children by childcare staff [19]. However, the staff in that study seemed to talk about healthy nutrition considerately more often than childcare staff in the current study: In the study of Sisson, 85.8% of the staff talked about healthy eating most to all the time [19], while only 34.8% of the staff in the current study often talked about healthy nutrition. This difference might have to do with differences between the countries in which the studies were conducted (*i.e.*, the U.S. *vs.* the Netherlands), with regard to staff’s knowledge of healthy nutrition. Staff’s knowledge about feeding has been shown to be an important predictor of whether they educate children about nutrition at childcare. In addition, knowledge about feeding is a predictor of desirable childcare staff practices in general [20]. We, therefore, recommend healthy nutrition to be included in education for all childcare workers. The low use of instrumental feeding (using food to control behavior) in childcare is consistently seen throughout studies [19,25], including the current study. However, it contradicts the regular use of this practice by parents (e.g., [38]). Previous research has shown that instrumental feeding can have detrimental effects on children’s food preferences, dietary intake and weight status (e.g., [34,39,40]).

The current study showed that when staff explained what they were doing during food preparation, children ate more fruit. In addition, when children were allowed to help prepare the food, the children ate less sweet snacks. Several studies with regard to child involvement in meal preparation at home have also shown a positive association with children’s intake of [41] and preference for [42] healthy foods. Involving children in food preparation has been shown to make them feel positive and in control [41], it increases eating enjoyment [43], and it increases self-efficacy for selecting healthy foods [42]. Thus, involving children in food preparation can have a positive influence on children, and the current study shows this is also the case at childcare. Involving children in food preparation is suggested to contribute to a positive context for eating and introducing new foods [41], which is important for developing food likes and dislikes [44]. However, more than 40% of the staff in the current study never or seldom involved children in food preparation. Perhaps childcare staff considers letting the children prepare food themselves as not feasible, because of for instance time constraints, the young age of the children, and the large number of children. The studies regarding child involvement in meal preparation described above, all regarded older children (>6 years) [41,42,43]. However, as an alternative, staff could tell the children what they are doing during meal preparation, as this was also positively associated with healthy food intake in the current study. Moreover, the tasks could be adjusted to the age of the child to increase feasibility: younger children could be involved in setting the table and washing fruit or vegetables, while older children could make their own sandwich or help with preparing the meals.

When staff gave children food without asking them whether they wanted any, the children ate less sweet snacks. Hughes and colleagues [27] found that if childcare staff gave or offered children multiple servings, children consumed more vegetables. In addition, in the current study, children ate more vegetables when staff encouraged children to continue eating (e.g., by obligating children to clear their plate). Although we did not explicitly assess the food practices in relation to *specific* meals or food items in the current study, we think staff might have linked giving food without asking mainly to main meals, not to snacks. The children in the current study might thus have eaten more at the main meals due to the staff giving them food without asking, causing them to eat less snacks. More research is however needed to confirm this hypothesis. The lack of other associations between the practices and vegetable intake, can probably be attributed to the low average intake of vegetables in the sample, due to the fact that most Dutch childcare centers do not serve warm meals.

Findings with regard to the effect of modeling of food intake by childcare staff are contradictory. A previous paper showed that children consumed more energy and dietary fiber if staff ate together with them [28]. The current study showed that children ate more sweet snacks if staff ate together with them. Another study found no effect of silent modeling of food intake by teachers on food acceptance by preschoolers [29]. However, the conditions under which this modeling takes place seem to be of importance. When preschool teachers added an enthusiastic statement about the food they were eating (e.g., “Mmm! I love mangos!”) to the modeling, the modeling was effective in increasing children’s food acceptance [29]. In line with this, talking about healthy foods with the children has been found to be positively associated with dietary fiber intake at childcare in a previous study [10], although this could not be confirmed by the current study. More research is needed to examine the effects of childcare staff modeling on children’s food intake, and under which circumstances these effects can be established. In addition to what staff says about the foods they eat, other contextual factors might also be of importance, such as what other children in the group do and say during that meal. Hendy and Raudenbush [29] have for instance shown that especially girls are very susceptive to the influence of a picky peer, overruling the positive effects of an enthusiastic teacher model.

There were several limitations of the current study that need to be noted. The current study was cross-sectional, therefore inferences regarding causality cannot be made. Dietary intake at childcare was observed and recorded by childcare staff. This possibly introduced bias because staff under or over reported intake of certain foods. The questionnaires regarding staff’s food practices were filled out together with the dieticians, which could increase social desirability bias. In addition, the external validity of the study finding might be limited as dietary intake was assessed on only two days and childcare centers were included on a voluntary basis. The centers included in the study might thus be relatively ‘healthy’ childcare centers. Moreover, only one of the staff members per group filled out this questionnaire. Other staff members not participating in the study might have used different food practices. Another limitation is the fact that macro level influences (e.g., national laws and regulations) were not taken into account. Furthermore, because validated questionnaires to assess childcare staff’s food practices are as yet not available, a questionnaire was created specifically for the current study, based on previous studies. This questionnarie was not validated. The lack of validated questionnaires to assess childcare staff practices is a large contrast with the abundance of questionnaires to assess parental food practices, and reflects the gap between our knowledge regarding social influences in both settings (home and childcare). We therefore call for systematic development and validation of questionnaires to assess childcare staff food practices. A strength of the current study was the relatively large sample size compared to previous studies on the same topic (N = 398), which provided sufficient statistical power to correct the analyses for the childcare center a child was attending, and thus multi-level structure of the data. Children’s dietary intake was observed instead of relying on questionnaires. Furthermore, the analyses were corrected for children’s age and gender, taking possible confounding by these variables into account. However, other possibly important factors such as peer modeling and children’s weight status were not taken into account in the current study. The importance of these factors and their interaction with the influence of food practices should be further examined in future studies. We know from the parenting literature that the same food practices might have different effects on different children, depending on factors such as their temperament and BMI [31,32,45].

In addition, more research into the determinants and predictors of the use of childcare staff’s food practices is needed. A study by Lanigan [20] for instance showed that childcare providers’ efficacy, feeding knowledge and misconceptions are important determinants of the food-related practices they use. Dev and colleagues [23] recently showed that factors such as providers’ own intentions to lose weight, their concerns about the children’s weight and their race predict which practices they use. Knowledge regarding these predictors can be used to inform future interventions to facilitate healthy food practices and dietary intake in the childcare setting.

## 5. Conclusions

The current study examined the use of food practices by childcare staff and their association with children’s dietary intake. In view of the low water consumption (only 2.0% of the children drank water) and vegetable consumption (mean 9.6 g), future efforts to improve children’s nutrition in childcare could focus on these behaviors. This is in line with increased attention for promoting water consumption in schools (e.g., [46,47]). The low vegetable intake can probably be attributed to the fact that Dutch childcare centers generally do not provide a warm meal. We, therefore, recommend providing vegetables at lunch and snack times. The current study further showed the importance of several childcare staff food practices for children’s dietary intake. Although more research is needed to examine the conditions under which these practices can have a positive impact on children’s nutrition, especially involving children in meal preparation at childcare seems to hold promising results to prevent obesity in the young children.
